# COVID-19 severity: does the genetic landscape of rare variants matter?

**DOI:** 10.3389/fgene.2023.1152768

**Published:** 2023-06-29

**Authors:** Maryam B. Khadzhieva, Alesya S. Gracheva, Olesya B. Belopolskaya, Dmitry S. Kolobkov, Darya A. Kashatnikova, Ivan V. Redkin, Artem N. Kuzovlev, Andrey V. Grechko, Lyubov E. Salnikova

**Affiliations:** ^1^ Federal Research and Clinical Center of Intensive Care Medicine and Rehabilitology, Moscow, Russia; ^2^The Laboratory of Ecological Genetics, Vavilov Institute of General Genetics, Russian Academy of Sciences, Moscow, Russia; ^3^The Laboratory of Molecular Immunology, Dmitry Rogachev National Medical Research Center of Pediatric Hematology, Oncology and Immunology, Moscow, Russia; ^4^The Department of Population Genetics, Vavilov Institute of General Genetics, Russian Academy of Sciences, Moscow, Russia; ^5^ The Resource Center “Bio-bank Center”, Research Park of St. Petersburg State University, St. Petersburg, Russia; ^6^ The Laboratory of Genogeography, Vavilov Institute of General Genetics, Russian Academy of Sciences, Moscow, Russia

**Keywords:** severe COVID-19, whole-exome sequencing, omnigenic model, rare variants burden, intolerant genes, genes associated with primary immunodeficiencies

## Abstract

Rare variants affecting host defense against pathogens may be involved in COVID-19 severity, but most rare variants are not expected to have a major impact on the course of COVID-19. We hypothesized that the accumulation of weak effects of many rare functional variants throughout the exome may contribute to the overall risk in patients with severe disease. This assumption is consistent with the omnigenic model of the relationship between genetic and phenotypic variation in complex traits, according to which association signals tend to spread across most of the genome through gene regulatory networks from genes outside the major pathways to disease-related genes. We performed whole-exome sequencing and compared the burden of rare variants in 57 patients with severe and 29 patients with mild/moderate COVID-19. At the whole-exome level, we observed an excess of rare, predominantly high-impact (HI) variants in the group with severe COVID-19. Restriction to genes intolerant to HI or damaging missense variants increased enrichment for these classes of variants. Among various sets of genes, an increased signal of rare HI variants was demonstrated predominantly for primary immunodeficiency genes and the entire set of genes associated with immune diseases, as well as for genes associated with respiratory diseases. We advocate taking the ideas of the omnigenic model into account in COVID-19 studies.

## Introduction

Since the first pandemic outbreak of coronavirus in 2019 (COVID-19), more than 664 million cases of COVID-19 and more than 6.7 million deaths have been reported (https://coronavirus.jhu.edu, assessed 9 January 2023). The clinical presentation of SARS-CoV-2 infection is highly variable, ranging from asymptomatic infection to severe disease with respiratory failure, overactive immune response, and death occurring in about 0.5%–1% of infections (Salzberger et al., 2021). Known risk factors for severe disease course include demographic characteristics (male sex, older age, and ancestry) and chronic diseases such as obesity, as well as cardiovascular, renal, and respiratory disease (Yang et al., 2020; O'Driscoll et al., 2021; Sokologorskiy et al., 2022), but these factors do not fully explain differences in clinical severity (Duman et al., 2022; Nhung et al., 2022; Petersen et al., 2022).

An individual’s genetic background influences susceptibility to infectious diseases and the severity of their course (Kwok et al., 2021). The GWAS meta-analysis of the worldwide COVID-19 Host Genetics (HG) Initiative identified 23 loci with minor allele frequencies ranging from 0.003 to 0.66, of which seven loci influenced susceptibility to SARS-CoV-2 infection, and 16 loci, including loci involved in inflammation or innate immunity (e.g., the *OAS1*/*OAS2*/*OAS3* gene cluster, *IFNAR2*, *DPP9*, *TYK2*, *SFTPD*, and *MUC5B*) were strongly associated with critical disease (COVID-19 Host Genetics Initiative, 2022). Measuring the association of rare genetic variants with COVID-19 is more challenging (Butler-Laporte et al., 2022). Although variants with a large effect on COVID-19 are likely to be rare, most rare variants are likely to have little effect on COVID-19 severity (Ganna et al., 2018). Most studies of genetic associations of rare variants have insufficient test power, and the resulting associations are not reproducible in independent cohorts (Zhang et al., 2020; Kosmicki et al., 2021a). The largest meta-analysis of rare variants performed by the COVID-19 HG project team showed one exome-wide significant association with severe COVID-19 for rare deleterious variants in the *TLR7* gene (Butler-Laporte et al., 2022).

In some chronic diseases, exome sequencing to test for enrichment of high-impact (HI) (https://grch37.ensembl.org/info/genome/variation/prediction/predicted_data.html) and/or damaging missense variants assessed as qualifying variants (QVs) in patients compared to controls was performed not only at the gene level but also in large specific gene sets. A significant excess of rare QVs in genes intolerant to loss of function or missense variants was observed in patients with psychiatric disorders (Ganna et al., 2018; Feng et al., 2019; Zoghbi et al., 2021) and amyotrophic lateral sclerosis (Farhan et al., 2019). A higher burden of QVs in patients with psoriasis compared to controls was found in the immune disease-related gene set (Xu et al., 2021). An enrichment in deleterious ultra-rare variants in gene sets previously associated with epilepsy was observed in epilepsy-affected individuals compared to controls (Feng et al., 2019). Large sets of genes with QVs have never been tested in relation to infectious diseases and critical conditions, although this approach may be promising because the recovery from acute infection requires the coordinated action of many genes (Talla et al., 2021).

The relationship between some or many genetic variations and complex traits is described by two main hypotheses: polygenic and omnigenic. In the polygenic model, disease-associated variants are combined into key pathways that determine disease manifestation and progression (Marouli et al., 2017). According to the omnigenic model, disease can be caused by a huge number of variants in most of the genome because the regulatory gene networks are highly interconnected. Through regulatory networks, associative signals from many so-called peripheral genes are transmitted to a much smaller number of core genes directly related to the disease (Boyle et al., 2017). Thus, all genes with regulatory variants expressed in disease-relevant cells can affect the function of the major genes associated with the disease. The omnigenic model was developed based on data from genome-wide studies for common genetic variants, but subsequent work has shown that a similar approach holds true for rare variants (Pullabhatla et al., 2018; Scelsi et al., 2021), which, while consistent with life, may have small effect sizes but be associated with more severe and/or earlier diagnoses (Wray et al., 2018). Although the concept of a set of core genes and a rigid dichotomy of core and peripheral genes is still debated (Wray et al., 2018; Iakoucheva et al., 2019), the general idea of a wide dispersion of genetic contributions to disease development due to the interconnectedness of biological systems seems widely accepted (Giral et al., 2018). In fact, the poly- and omnigenic models do not contradict each other, and recent studies have shown broad compatibility between the two models (Lombardo et al., 2018; Vuckovic et al., 2020; Zhu et al., 2021).

In this study, we performed whole-exome sequencing in 86 Russian patients with severe or mild/moderate COVID-19. Given the omnigenic hypothesis, we aimed to characterize the entire exomic landscape of rare genetic variants at different disease severity. We also set out to compare the burden of rare variants in patient groups stratified by sex, age and comorbidities. Our subsequent analyses of COVID-19 phenotypes focused on testing the enrichment of QVs in sets of genes, particularly intolerant to the variants under consideration and biologically relevant to COVID-19.

## Materials and methods

### Patients and clinical data

The study included 86 patients diagnosed with COVID-19 who were hospitalized at the Moscow Regional Scientific and Clinical Institute named after M. F. Vladimirsky, the Moscow Clinical Center for Infectious Diseases “Voronovskoe” and the City Clinical Hospital of the Moscow Department of Health named after V.P. Demikhov from April 27 to 28 November 2020. The exclusion criteria were as follows: patients with terminal incurable diseases, immunodeficiency, long-term use of corticosteroids, pregnancy, alcoholism, drug addiction, and HIV. Upon admission, all patients underwent a PCR test for the SARS-CoV-2 virus from nasopharyngeal smears. The diagnosis and severity of the course of the disease were established in accordance with international recommendations for the prevention, diagnosis and treatment of new coronavirus infection (COVID-19) (World Health Organization, 2020) and “Temporary clinical guidelines on prophylaxic, diagnosis and treatment of COVID-19–2020” of the Russian Ministry of Health (ВМР_COVID-19_V17. pdf (minzdrav.gov.ru)). All patients were divided into two groups. Severe COVID-19 patients included patients with severe/extremely severe course (57 patients), while non-severe COVID-19 patients included patients with mild/moderate course (29 patients) (Supplementary Table S1). The diagnosis of pneumonia was made according to provisional clinical guidelines (ВМР_COVID-19_V17. pdf (minzdrav.gov.ru)), taking into account the results of chest computed tomography with an assessment of the severity of lung damage using a point scale (Supplementary Table S1), as well as clinical and laboratory data described elsewhere (Kashatnikova et al., 2022). The diagnosis of sepsis has been revised according to the 2021 version of the Sepsis Survival Campaign guide (Evans et al., 2021). The diagnosis of ARDS was based on the Berlin definition (Force et al., 2012). The Ethics Committee of the Federal Research and Clinical Center of Intensive Care Medicine and Rehabilitology approved the study; all included patients or their legal representatives signed an informed consent.

### Exome sequencing and variant calling

DNA was isolated from blood using Qiagen DNA blood mini kit. Fifty samples were sequenced at Genomed (Moscow, Russia). DNA was fragmented and barcoded using Swift 2S^®^ Turbo DNA Library Kit. Enrichment was performed using Twist Human Core Exome (https://www.twistbioscience.com/products/ngs/fixed-panels/human-core-exome). Sequencing was implemented on Illumina Hiseq X Ten platform with 150 bp paired-end reads. Thirty-six samples were sequenced at Resource Center “Bio-bank Center”, the Research Park of St. Petersburg State University (St. Petersburg, Russia). Samples were prepared for sequencing with Illumina TruSeq DNA Exome kit (https://www.illumina.com/products/by-type/sequencing-kits/library-prep-kits/truseq-exome.html) and sequenced on HiSeq2500 and HiSeq4000 platforms with 90 bp paired-end reads.

The sequencing data of 86 samples in FASTQ format were analyzed. Reads were aligned to human reference GRCh38. p13 using Burrows-Wheeler Alignment Tool (bwa https://bio-bwa.sourceforge.net/bwa.shtml). The quality control of sequencing results performed in the FastQC program (Andrews, 2010) showed high quality of readings and the presence of an insignificant number of adapter sequences. Data preparation was carried out by the programs of the GATK package (Van der Auwera and O'Connor, 2020), namely, for each sample, the source FASTQ files were converted to bam files without alignment by the FastqToSam utility, adapter sequences were labeled using MarkIllumina Adapters. Mapping of readings to the reference genome was carried out using BWA MEM (Li, 2013), after which the marking of PCR duplicates by the MarkDuplicates program was carried out. The target regions in the analysis were the genome regions common to the two sets that were used for sample preparation. The quality of mapping results and targeted enrichment with target sequences was evaluated using the CollectHsMetrics program.

The search for variants in bam format files was carried out using the HaplotypeCaller program of the GATK package, a filter was applied to the results using the VariantFiltration program of the GATK package. Only variants within the target regions were analyzed. The filtering conditions included a value of QD (Variant Confidence/Quality by Depth) of at least 2.0, a value of FS (Phred-scaled *p*-value using Fisher’s exact test to detect strand bias) of ≥60 and Strand Odds Ratio (SOR) < 3 (for indels FS ≥ 200 and SOR <10), a value of MQ (RMS Mapping Quality) of at least 40, a value of MQRankSum of at least −12.5, a value of ReadPosRankSum (Z-score from Wilcoxon rank sum test of Alt vs. Ref read position bias) at least −8.0. Variants were required to pass GATK’s standard variant quality score recalibration (VQSR) threshold. We removed variants previously identified as problematic by ExAC (Lek et al., 2016), GnomAD (Karczewski et al., 2020), or EVS (http://evs.gs.washington.edu/EVS/HelpDescriptions.jsp). Variant calls were required to have at least 10x coverage. For heterozygous genotypes, the alternative allele ratio (allelic balance) was set to be ≥25%. Site-coverage harmonization between samples of patients with severe COVID-19 and non-severe COVID-19 was performed by removal of all variants with a greater than 7% absolute difference in 10-fold coverage (Petrovski et al., 2017). Average sequencing depth was 75.22 ± 56.59 (mean ± SD) in patients with severe COVID-19 and 73.39 ± 56.71 in patients with non-severe COVID-19.

### Variant annotation

The annotation of the variants was carried out by the SnpSift (Cingolani et al., 2012a), SnpEff (Cingolani et al., 2012b) and FAVOR programs (Zhou et al., 2023) using the dbSNP, ClinVar, and population databases GnomAD (Karczewski, et al., 2020), 1000G (1000 Genomes Project Consortium, 2015) and TopMed (Taliun et al., 2021). Variants with presumably disruptive impact in the protein were classified as HI. They included splice acceptor variants, splice donor variants, stop gained, frameshift variants, stop lost, and start lost variants. Missense variants were defined as potentially pathogenic using the rare exome variant ensemble learner (REVEL) tool with a recommended threshold of >0.5 to classify the variant as “harmful” (Ioannidis et al., 2016). We also used the missense tolerance ratio (MTR) tool, which detects regional intolerance to missense variations. As recommended by the authors, we applied the conservative threshold of MTR FDR <0.1 (Silk et al., 2019). Our analysis focused on rare variants, which were filtered according an alternative allele frequency (AF) in the Genome Aggregation Database (GnomAD), 1000 G and TopMed databases. Since our sample was rather small, in order to combine our results with the available literature data and to minimize possible bias from sample size, we focused not on the internal AF, but on AF from these databases. The variants were divided by AF into three main bins with AF ≤0.001 to ≤0.01, with AF <0.001 and without AF data in population resources, i.e., not present in any of the considered population databases.

### Gene set curation

Because the omnigenic hypothesis suggests that genes with regulatory variants in at least one disease-relevant tissue are likely to influence disease risk, we compiled a list of eGenes (genes whose expression levels are associated with at least one genetic variant) using data from the GTEx V8 release with a threshold *Q* value (*p*-value with FDR correction) of 0.05 (https://gtexportal.org/home/datasets).

To create sets of genes intolerant to missense variants and loss of function, i.e., HI variants, we used the recommended threshold values pLI >0.9 and missense Z score >3.09 based on constraint metrics from gnomAD v2.1 (https://storage.googleapis.com/gnomadpublic/release/2.1.1/constraint/gnomad.v2.1.1.lof_metrics.by_gene.txt.bgz). Missense and loss-of-function tolerant gene sets comprised genes with missense Z < 1 and pLI <0.001, respectively (Zoghbi et al., 2021). We also generated gene sets of genes essential for life (Dickinson et al., 2016), genes of primary immunodeficiencies (PID) from the 2022 Update on the Classification from the International Union of Immunological Societies (IUIS) Expert Committee (Tangye et al., 2022), cytokine genes encoding proteins with cytokine/chemokine activity and cytokine/chemokine receptor activity (Salnikova et al., 2020) and the updated list of genes linked to SARS-CoV-2 infection and/or COVID-19 disease from the GENCODE project (https://www.gencodegenes.org/human/covid19_genes.html#, accessed 12 December 2022). Our next approach was to analyze the lists of genes associated with human diseases affecting certain organs and systems. We used our previously constructed gene list of disease genes described in detail elsewhere (Kolobkov et al., 2022). Shortly, we generated a set of 9,972 genes from five gene-phenotype databases (OMIM, ORPHANET, DDG2P, DisGeNet and MalaCards) and a report of the IUIS. A gene was considered to be associated with a specific system (e.g., immune, respiratory, nervous) according to the classification of any of the resources used.

### Principal component analysis

Since some of the results may be population-specific, we explored the cohort’s population structure by projecting it onto 1,000 Genome’s principal component space built using the intersection of variants between the two datasets. The joint analysis with the 1,000 Genomes dataset was conducted using PLINK2 (https://doi.org/10.1186/s13742-015-0047-8). The visualization was conducted using the Plotly library in Python.

### Association analysis

Association analysis of rare variant series was carried out using unadjusted two-sided Cochran–Mantel–Haenszel (CMH) test, which generates a combined *p*-value and odds ratio (McDonald, 2009; Zoghbi et al., 2021). The CMH statistic extends Fisher’s exact criterion beyond stratified contingency tables 2 × 2 to test whether the common odds ratios across strata is equal to 1 (Rahardja et al., 2016). This test was preferred to logistic regression due to its robustness and the lack of inflation of test statistics in situations with a small number of carriers (Cirulli et al., 2020). A dominant model of inheritance was considered. In order to account for the possible influence of covariates such as gender and age on the associations identified, we conducted a subgroup analysis. We applied an experiment-wise *p*-value threshold of 5.21 × 10^−4^ to account for multiple testing (0.05/96 comparisons tested).

### Gene-based burden analysis

In order to identify genes with mutational burden statistically different between severe and non-severe COVID-19 patients we collapsed qualifying variants (QVs) at the gene level. We considered three sets of rare QVs combined in the set of variants from bins with AF <0.001 and no AF data. Qualifying variants included HI variants, missense variants with REVEL >0.5 and HI + missense variants with REVEL >0.5. In haploinsufficient (HIS) genes sensitive to decreased gene dosage, any copy of a gene containing a harmful variant will cause a detrimental effect on gene function. DECIPHER (https://www.deciphergenomics.org/about/downloads/data) provides haploinsufficiency scores which are determined based on predicted HIS probabilities. Scores in the range 0%–10% mean a higher probability of a gene to be HIS, while scores in the range 90%–100% mean that a gene is unlikely to be HIS. In HIS genes (with HIS scores ≤10%), we examined the same three sets of QVs as described above but without limiting to AF. For each gene, if any individual in the severe COVID-19 group has ≥1 QVs in the gene, the group will count one, otherwise it will count 0; the same for the non-severe COVID-19 group. Then a Fisher’s exact test with Bonferroni correction (Cirulli et al., 2020) (16,816 genes, six comparisons, significant *p*-value <4.96 × 10^−7^) was performed to see if there is a difference between the two groups.

## Results

### Clinical and demographic characteristics

A total of 86 unrelated patients with COVID-19 were included in the current study. All patients were unvaccinated, since blood samples were collected in Russia in the era before vaccination, until 2021. The main demographic and clinical characteristics of patients are summarized in Table 1. The group with severe COVID-19 included 57 patients with severe/extremely severe course, while the group with mild COVID-19 included 29 patients with mild/moderate COVID-19. The two groups did not differ in demographic indicators and pre-existing conditions. As expected, there were differences in the number of patients admitted to the ICU, requiring ventilation and having a more severe degree of lung parenchyma lesion on CT. All deceased patients had severe COVID-19 disease.

**TABLE 1:** Demographic and clinical characteristics of the COVID-19 patients.

Characteristics	Severe COVID-19 (*n* = 57)	Non-severe COVID-19 (*n* = 29)	*p*-value^a^
**Demographic**			
Gender (Males)	32 (56.14%)	18 (62.07%)	0.649
Age (Mean ± SD)	59.6 ± 13.38	58.62 ± 22.86	0.537 (MWU)
Age (Range)	27–85	24–96	—
**Pre-existing conditions**			
No comorbidity	1 (1.75%)	5 (17.24%)	0.015
Hypertension	40 (70.18%)	18 (62.07%)	0.474
Dyslipidemia	2 (3.51%)	0 (0%)	0.548
Hypercholesterolemia	1 (1.75%)	1 (3.45%)	1.000
Type 2 diabetes	20 (35.09%)	10 (34.48%)	1.000
Obesity	18 (31.58%)	8 (27.59%)	0.806
Cardiovascular disease	30 (52.63%)	12 (41.38%)	0.367
Neurological disease	5 (8.77%)	7 (24.14%)	0.096
Respiratory disease	10 (17.54%)	7 (24.14%)	0.569
oCOPD	1 (1.75%)	0 (0%)	1.000
oAsthma	1 (1.75%)	1 (3.45%)	1.000
Digestive/liver disease	14 (24.87%)	7 (24.14%)	1.000
Cancer^b^	3 (5.26%)	2 (6.9%)	1.000
Kidney disease	10 (17.54%)	2 (6.9%)	0.323
**Management and clinical course**			
ICU	55 (96.49%)	12 (41.38%)	**1.6 × 10** ^−8^
Ventilatory support			**2.3 × 10** ^**−5**^ (LR)
Invasive ventilation	30 (52.63%)	1 (3.45%)	
Non-invasive ventilation	1 (1.75%)	0 (0%)	
Without ventilatory support	26 (45.61%)	28 (96.55%)	
Hospitalization (mean ± SD of days)	17.95 ± 10.79	13.7 ± 5.9	0.265 (MWU)
Chest CT severity scoring			**6.8 × 10** ^**−4**^ (LR for scores 0–4)
0	1 (1.75%)	1 (3.45%)	
1	4 (7.02%)	9 (31.03%)	
2	7 (12.28%)	9 (31.03%)	
3	23 (40.35%)	10 (34.48%)	
4	17 (29.82%)	0 (0%)	
No data	5 (8.77%)	0 (0%)	
Pneumonia	57 (100%)	28 (96.55%)	0.337
ARDS	18 (31.58%)	3 (10.34%)	0.035
Sepsis/septic shock	6 (10.53%)	0 (0%)	0.093
Outcome			
Recovered	23 (40.35%)	29 (100%)	**2.7 × 10** ^−8^
Deceased	31 (54.39%)	0 (0%)	
No data (transfer to another hospital)	3 (5.26%)	0 (0%)	

^a^ The exact two-tailed Fisher test unless otherwise indicated in parentheses.

^b^ Complete remission [Severe COVID, group: lung cancer (1), kidney cancer (1), uterine cancer (1); non-severe COVID, group: lung cancer (1), rectal cancer (1)]. LR, the likelihood ratio χ2 test; MWU, the Mann–Whitney *U* test. Significant results with a *p*-value threshold of 2.1 × 10^−3^ to account for multiple testing are highlighted in bold.

### Overview of genetic landscape

**FIGURE 1:**
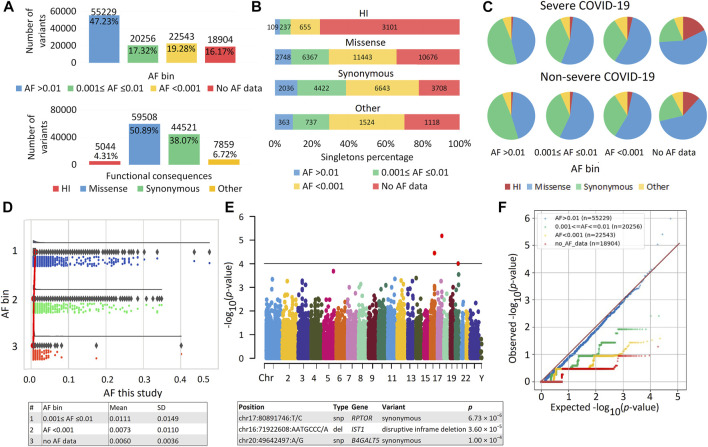
| Exome data. **(A)** Distribution of variants by allele frequency (AF) bins according to population databases and different functional consequences. **(B)** Distribution of variants by number of singletons. **(C)** Proportion of variants with different AF and functional consequences in patients with severe and non-severe COVID-19. **(D)** Raincloud plot to compare internal AF with data from population databases, including three subplots for different AF bins with cloud for data kernel density—half violin, umbrella for boxplot, rain for data points below the cloud and flash for connection lines between means. The mean and SD of the internal AF for the AF bins are shown in the table. **(E)** Manhattan plot of association p values for COVID-19 Severity. Genes passing the threshold 1.0 × 10^−4^ are displayed in the table. The significant exome-wide *p*-value is 4.28 × 10^−7^ (Bonferroni correction: 0.05/116,932). Manhattan plot and raincloud plot were constructed using https://www.bioinformatics.com.cn/en, a free online platform for data analysis and visualization. **(F)** Quantile-Quantile (QQ) plot of association results for variants from different AF bins.

A total of 2,504,482 genetic variants were identified in our sample. After filtering, 1,983,390 variants remained; the number of variants per person was 28,133.56 ± 914.86. The number of unique variants, i.e., variants with unique identifiers (position and alleles), was 116,932. These variants were located in 16,816 different genes, among which 95.02% were eGenes expressed in an average of 18 tissues (Supplementary Table S2). The distribution of variants by AF and various functional consequences is shown in Figure 1A. Common variants (AF >0.01) were the most numerous; missense variants predominated among variants with different functional consequences. Among HI variants, the majority of variants (81%) were in the heterozygous state, and more than half were represented by frameshift variants (Supplementary Figure S1A). HI variants without AF data included the greatest number of singletons (Figure 1B). Maximum differences between patients with severe *versus* non-severe COVID-19 were also observed in the proportions of these variants (*p* = 6.90 × 10^−16^) (Figure 1C; Supplementary Table S3). As expected, as the frequency of genetic variants decreased, so did the sets of overlapping genes in the two patient groups (Supplementary Table S4). The number of HI variants per individual was 217.39 ± 100.51, which is consistent with the literature, according to which the number of HI variants per individual can range from 100 to 800 variants (MacArthur et al., 2012; 1000 Genomes Project, 20151000 Genomes Project Consortium, 2015; Johnston et al., 2015; Lek et al., 2016). We compared the AF in our sample with frequencies from population databases. The internal frequencies in the entire sample (Figure 1D) and in the major variant types (HI, missense, and synonymous) (Supplementary Figure S1B) were consistent with the population data. The frequencies of variants in bins AF < 0.001 and without AF data were similar, but in the latter case the scatter of the data was smaller. There were no significant associations of individual variants with COVID-19 severity (Figure 1E). The quantile-quantile (Q-Q) plot showed deflation of the observed *p* values, which means that our sample size is insufficient to analyze and interpret the results at the level of individual genetic variants (Figure 1F). Deflation increased in subsets of variants in different AF bins as AF decreased. These results reflect the low number of minor allele counts and an excess of singletons (Figure 1B) and are consistent with the literature (Kosmicki et al., 2021b).

Principal component analysis (PCA) showed that our cohort (sampled in Russia), with the exception of seven samples, clusters together with the European superpopulation (Supplementary Figure S2). We also compared AF in our sample with the data in the ESPOSITO database. This resource, developed by CEINGE researchers, contains data derived from whole-exome sequencing of more than 1,000 subjects infected with SARS-CoV-2. ESPOSITO-COVID (espocovid.ceinge.unina.it/) includes variant frequencies in individuals from southern Italy with asymptomatic and severe COVID-19 (D'Alterio et al., 2022). Of the 64,839 overlapping variants in our sample and in the ESPOSITO-COVID database, the vast majority of variants had similar frequencies (Supplementary Table S5). At significance thresholds of less than 0.05 and 0.01, there were 264 and 35 overlapping variants in both data sets, for which unidirectional effects occurred in 60.6% and 68.6% of cases, respectively.

### Distribution of rare and pathogenic/likely pathogenic (P/LP) variants between severe and non-severe COVID-19 groups

**FIGURE 2:**
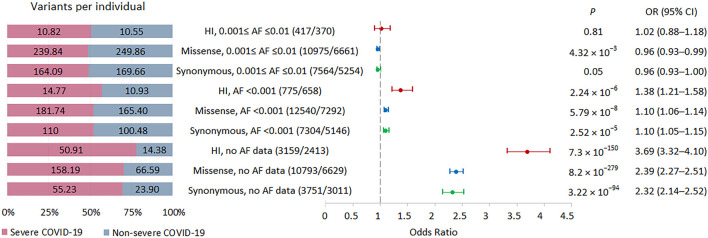
| Burden of rare HI, missense, and synonymous variants in 57 individuals with severe COVID-19 and 29 individuals with non-severe COVID-19. Odds ratios and horizontal bars denoting 95% confidence intervals are shown. The number of variants/number of genes for each set of variants is shown in parentheses. The left subplot shows the distribution of variants per individual using a 100% stacked bar chart. The absolute numbers of variants per individual for each set of variants are indicated. Significant *p*-value threshold is 5.21 × 10^−4^.

Our primary analysis focused on comparing the burden of rare HI, missense and synonymous variants in individuals with severe and non-severe COVID-19 (Figure 2). We did not limit the study to considering eGenes, since only a small part of the genes in our study were not eGenes, and, according to GTEx, the detection of eGenes strongly depends on the sample size (GTEx Consortium, 2017), so as the GTEx project develops, we can expect an expansion of the eGenes list. We found that individuals with severe COVID-19 had a significantly higher burden of variants belonging to bins with AF <0.001 and no AF data, while the signal was absent for less rare variants from bin with AF ≤0.001 to ≤0.01. The effect size was higher for the HI variants, then for the missense variants, and the smallest effect size was observed for the synonymous variants. Thus, the analysis of the entire set of exome variants showed a significant increase in the burden for variants without AF data, with a maximum effect size for HI variants that was 3.5 times higher per person in severe compared to non-severe COVID-19 patients.

Next, we repeated this analysis by excluding all singletons (Supplementary Figure S3). In a significantly reduced sample, the results were essentially unchanged. We observed a significantly higher burden of HI variants related to bin with AF <0.001 and HI and missense variants without AF data in severe patients compared with non-severe COVID-19 patients.

As PCA showed that seven samples from our cohort did not cluster together with the rest, we removed them and repeated the association analysis without them (Supplementary Figure S4). The results were consistent with those for the entire sample.

We also examined the distribution of P/LP variants from the ClinVar database (https://www.ncbi.nlm.nih.gov/clinvar/, assessed 12 December 2022) in patients from the two groups. We found 175 overlapping P/LP variants in 167 genes whose distribution did not differ between patients with different COVID-19 severity (*p* = 0.15, OR = 0.88, 95% CI 0.73–1.04).

### Analysis in the subgroups of patients stratified by gender, age and comorbidities

**FIGURE 3:**
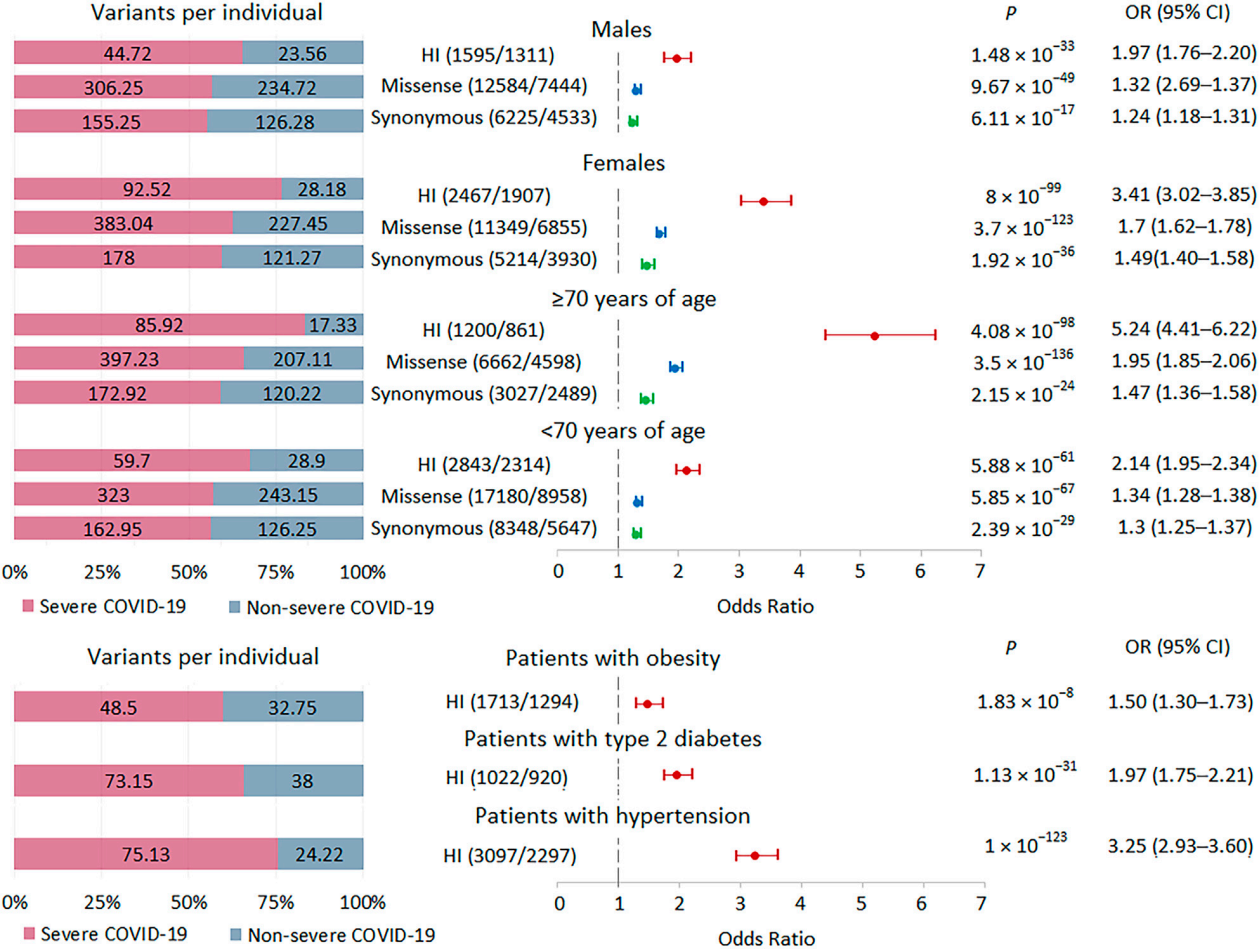
| Burden of rare HI, missense, and synonymous variants in subgroups with severe and non-severe COVID-19 formed by gender, age and comorbidities. Odds ratios and horizontal bars denoting 95% confidence intervals are shown. The number of variants/number of genes for each set of variants is shown in parentheses. The left subplot shows the distribution of variants per individual using a 100% stacked bar chart. The absolute numbers of variants per individual for each set of variants are indicated. Significant *p*-value threshold is 5.21 × 10^−4^.

Because the internal AF for variants assigned to bins with AF <0.001 and no AF data were similar (Figure 1C; Supplementary Figure S1B) and there was a significant increase in these variants in severe patients with COVID-19 compared to non-severe patients (Figure 2), we combined these variants into one set, termed rare variants, for subsequent analysis. Given the high burden of rare variants in severe COVID-19, we expect that analysis of relatively large subsets of patients, genes, and variants should also show enrichment of these variants in severe COVID-19, in which case we can compare effect sizes. We divided the groups of patients by gender and age. There were 32 men and 25 women with severe disease, 13 patients ≥70 years old and 44 patients <70 years old; there were 18 men and 11 women with non-severe disease, 9 patients ≥70 years old and 20 patients <70 years old. For all strata, the analysis showed highly significant results. The greatest effects were observed for HI variants, followed by missense variants. In the group with severe *versus* non-severe COVID-19, the burden of rare variants was higher in women than in men and in patients ≥70 years old compared to patients <70 years old (Figure 3). In the group with the highest association signal (age ≥70 years), the proportion of HI variants in patients with severe disease was 5 times higher than in patients with non-severe disease, who had the lowest number of HI variants per person, only 17.3.

We also examined the burden of rare HI variants in subgroups of patients with obesity, type 2 diabetes, and hypertension. We observed significant enrichment for these variants in all three subgroups of patients with severe COVID-19, with the lowest signal found in the obese subgroup (Figure 3).

### Rare variant burden in intolerant genes in severe COVID-19

**FIGURE 4:**
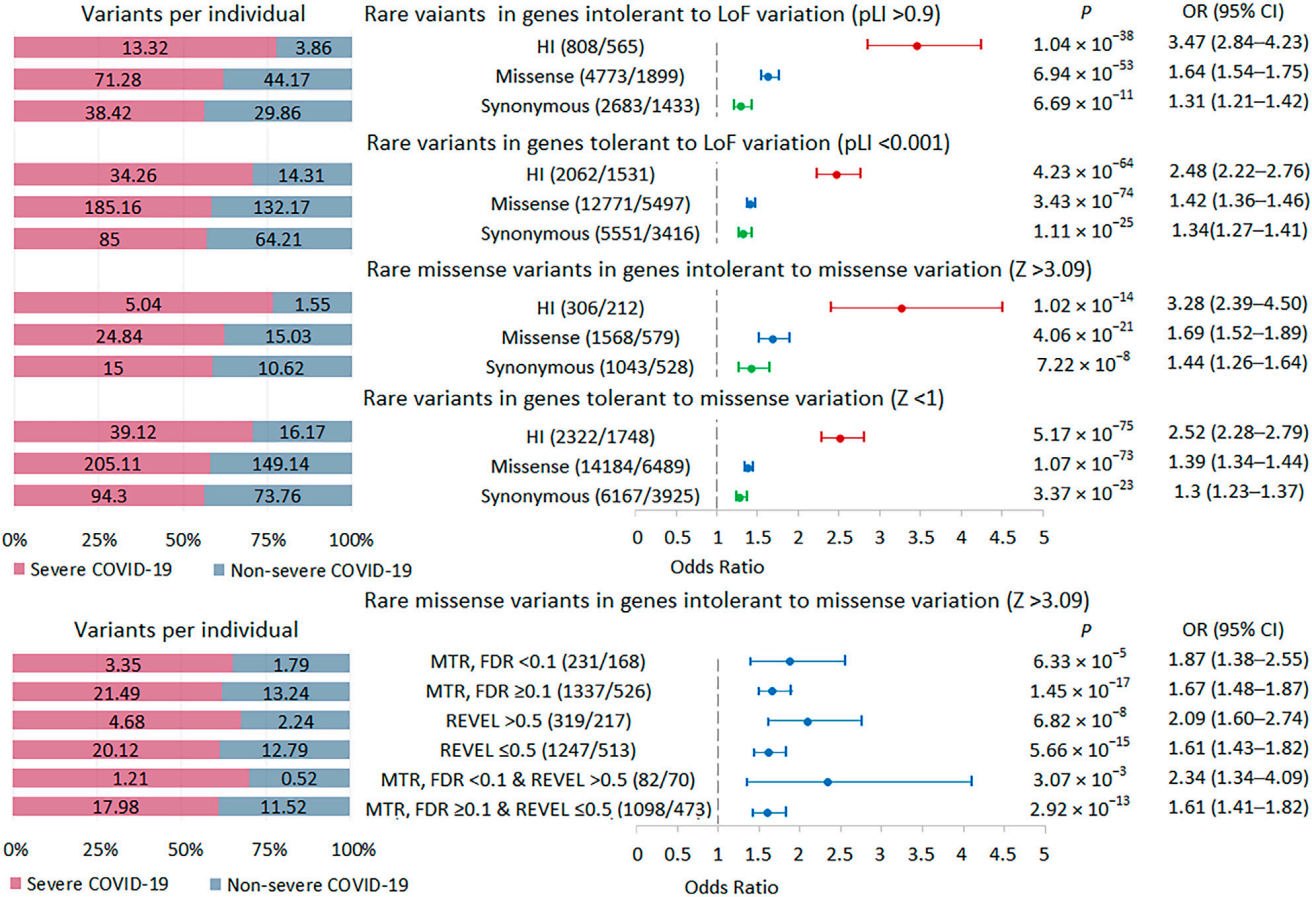
| Burden of rare variants in intolerant genes. Odds ratios and horizontal bars denoting 95% confidence intervals are shown. The number of variants/number of genes for each set of variants is shown in parentheses. The left subplot shows the distribution of variants per individual using a 100% stacked bar chart. The absolute numbers of variants per individual for each set of variants are indicated. Significant *p*-value threshold is 5.21 × 10^−4^.

We performed a burden test in two sets of intolerant genes: genes with a high probability of being loss-of-function intolerant, i.e., intolerant to HI variations (pLI score >0.9) and genes intolerant to missense variations (missense Z score >3.09) (Figure 4; Supplementary Table S2). The highest burden of rare HI variants was found in genes with a pLI score >0.9, followed by genes with a missense Z score >3.09. We used two tools to select potentially damaging missense variants in genes intolerant to missense variations. The missense tolerance ratio, MDR tool predicts the sensitivity of gene regions to missense variants, which may, for example, include regions corresponding to key protein domains (Silk et al., 2019). The rare exome variant ensemble learner, REVEL tool predicts variant pathogenicity, maximizing sensitivity and specificity to predict deleteriousness (Ioannidis et al., 2016). MTR FDR <0.1 and REVEL score >0.5 were used as thresholds. In missense-intolerant genes, we observed a stronger association signal for missense variants in gene regions with subgenic intolerance (MTR FDR <0.1) compared to missense variants in other gene regions (MTR FDR ≥0.1). We also found a higher burden of damaging missense variants (REVEL >0.5) compared to rare missense variants with a REVEL score ≤0.5. Combining these predictors further amplified the effect, but because the set of variants became much smaller, the statistical significance did not pass the experiment-wise *p*-value threshold 5.21 × 10^−4^. Summarizing these results, we can conclude that the more intolerant the genes are to variations and the more pathogenic these variations are, the larger the effect size when comparing groups of patients with different courses of COVID-19.

### Rare HI variant burden in selected gene sets in severe COVID-19

**FIGURE 5:**
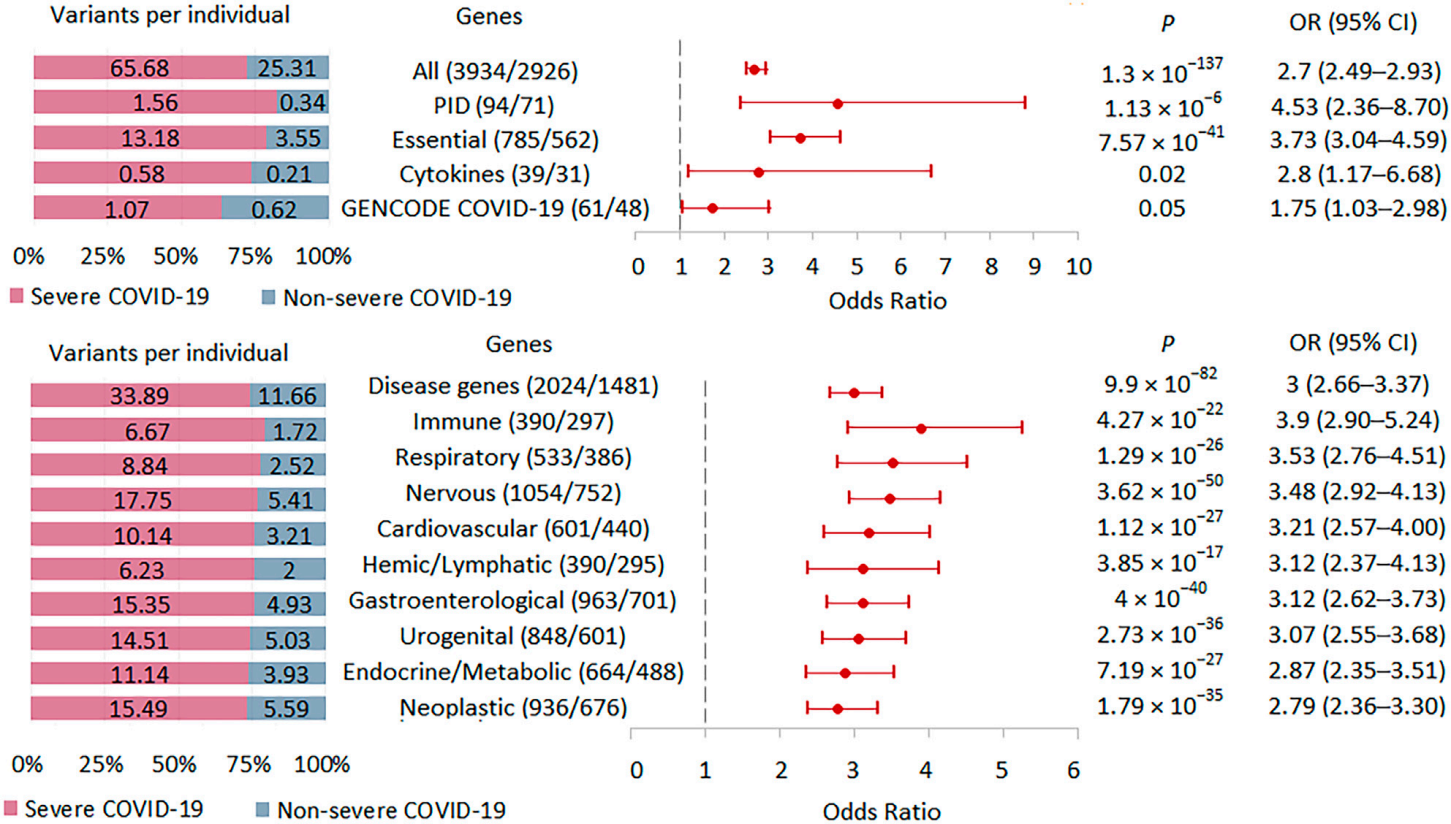
| Burden of rare HI variants in selected gene sets. Odds ratios and horizontal bars denoting 95% confidence intervals are shown. The number of variants/number of genes for each set of variants is shown in parentheses. The left subplot shows the distribution of variants per individual using a 100% stacked bar chart. The absolute numbers of variants per individual for each set of variants are indicated. Significant *p*-value threshold is 5.21 × 10^−4^.

We tested the burden of rare HI variants in several sets of genes that may be biologically important and interpretable in the context of the development and course of acute infection (Figure 5; Supplementary Table S2). The highest association signal was observed in the PID-related gene set (*n* = 71), followed by the signal observed in genes essential for life (*n* = 562). In these two sets of genes, the association signals were higher than in the entire set of genes with rare HI variants (*n* = 2926). The other two gene sets representing the cytokine network (*n* = 31) and the updated list of genes associated with SARS-CoV-2 infection and/or COVID-19 disease from the GENCODE project (*n* = 48) showed no significant enrichment, but we cannot rule out that this result is due to too small a sample size.

We also compared the burden of rare HI variants for gene sets associated with specific systemic diseases (Figure 5). The highest association signal was observed in the immune disease gene set (*n* = 297), which included 69 PID genes representing 23.2% of all immune genes. The second strongest association signal was in the respiratory disease gene set (*n* = 386), followed by the nervous system disease gene set (*n* = 752). Notably, the weakest effect was found for the neoplastic disease gene set (*n* = 676). Rare HI variants were 2.9, 3.9, and 2.8 times higher per person in severe compared with non-severe COVID-19 patients across the all-disease gene set, immune disease genes, and neoplastic disease genes, respectively.

### Gene-based qualifying variant collapsing analysis

We used a collapsing method to aggregate information across genes to amplify the signal from QVs (Supplementary Table S6). In none of the six series of rare HI variants, missense REVEL >0.5 and HI + missense REVEL >0.5 and the same series of QVs with any AF in the HIS genes, we found results with significance levels <1 × 10^−4^. No individual gene in our study can be considered as a candidate gene in a gene-level burden test.

## Discussion

In this study, we performed whole-exome sequencing to compare the distribution of rare genetic variations between patients with severe and mild/moderate course of COVID-19. In our cohort, patients with severe COVID-19 had an excess of rare variants at the whole-exome level. The burden of rare variants was higher in women than in men and in patients aged ≥70 years compared to patients aged <70 years. The excess of rare HI variants in severe COVID-19 was also observed in subgroups of patients with obesity, type 2 diabetes, and hypertension. Restriction to genes intolerant to HI or damaging missense variants increased the enrichment for these classes of variants in severe *versus* non-severe COVID-19. Analysis of rare HI variants in different gene sets showed the maximum burden effect size for PID genes followed by essential genes; among gene sets associated with diseases of various body systems, the highest enrichment levels were obtained for the immune and respiratory disease genes, while the least pronounced association signal was recorded for genes associated with neoplasia. Because major studies of host genetics in COVID-19 patients have focused on individual SNPs, genes, and pathway-level associations, which are limited to a relatively small number of predominantly “core” genes (Zhang et al., 2020; Kosmicki et al., 2021a; López-Rodríguez et al., 2022; Shcherbak et al., 2022) our study can be viewed as the first experimental work within the omnigenic model of polygenic heritability (Mathieson, 2021) in severe COVID-19.

The multifactorial and polygenic nature of COVID-19 has been extensively studied (Casanova et al., 2020). In addition to age, sex, and comorbidity, factors associated with this disease include socio-economic status and race/ethnicity (Webb Hooper et al., 2020). GWAS and exome sequencing studies have shown complex polygenic architecture of COVID-19. Immune system genes, primarily those involved in the interferon type I (IFN) signaling pathway, and genes related to lung function/respiratory disease were identified as key players in determining disease severity (Severe Covid-19 GWAS Group, et al., 2020; Zhang et al., 2020; COVID-19 Host Genetics Initiative, 2021; Pairo-Castineira et al., 2021). However, COVID-19 is a complex multisystem disease, and the complex traits are products of multiple genes that interact with each other in complex ways (Mathieson, 2021). Many more genes are expected to be involved in COVID-19 than the dozens reported thus far. These genes are likely to include a relatively small number of core genes and a much larger number of peripheral genes carrying both common and rare variants (Fallerini et al., 2022; Zguro et al., 2022).

In our sample, the burden of rare variants in severe *versus* non-severe COVID-19 decreased in the series HI > missense > synonymous variants. These results seem reasonable because HI variants include variants with presumably disruptive impact in the protein, missense variant pathogenicity depends on an amino acid change and a protein domain affected, while synonymous variants are often considered neutral. However, synonymous variants can also be functional because they can disrupt transcription, splicing, co-translational folding, mRNA stability, and can modulate gene expression by affecting transcription and splicing regulatory factors in protein-coding regions [reviewed in (Zeng and Bromberg, 2019)].

The magnitude of the difference between severe and non-severe COVID-19 was assessed in different age and sex groups and in subgroups of patients with common comorbidities of severe COVID-19, in particular, obesity, type 2 diabetes, and hypertension (Bailly et al., 2022)**.** The burden of rare most notably HI variants was higher in women than in men and in elderly patients compared to younger ones. Biological sex modulates the response to a number of infections, including SARS-CoV-2 infection, with men being at much greater risk of severe infection and adverse outcome than women (Takahashi et al., 2020). One explanation may be a stronger antiviral innate interferon response and higher levels of adaptive immunity to viral antigens in women (Peckham et al., 2020), because the *TLR7* gene, which is a sensor for viral RNA, is located on the X chromosome, thus contributing to sex bias in disease severity due to a gene dosage effect (Webb et al., 2019). Sex hormones can also affect sex-related differences in COVID-19 severity (Brandi, 2022). Given the gender disparity in the severity of COVID-19, an additional risk factor for the development of severe disease, that is, the burden of rare variants, should be stronger in women than in men. Regarding age, we observed an inverse association effect, i.e., an increased signal of the damaging rare variants in older patients, who are more susceptible to severe COVID-19. Age is the most important risk factor for the development of severe COVID-19 (Brodin, 2021). Ageing has a profound detrimental impact on almost all features of immune system. Presence of systemic basal mediators of inflammation increases with aging, and this occurs independently of acute immune challenges. This persistent, low-level, chronic inflammation is thought to be responsible for many of the chronic diseases associated with aging as well as a major contributor to immunosenescence. The serious consequences of SARS-CoV-2 infection are probably caused by a pathological hyperinflammatory response triggering tissue damage, vascular leakage, systemic cytokine storm and thrombosis. The elderly immune system may be vulnerable to such severe consequences, particularly because of defects in type I IFN production and signaling, impared T cell responses and dysregulated interactions between neutrophils and monocytes promoting excessive inflammation (Brodin, 2021). Because of abnormalities in regulatory pathways, an excess of rare variants can further weaken the aging immune system’s ability to resist infections, causing uncontrolled inflammation. In the elderly group, the association effect was enhanced not only because of the presence of a large number of QVs in severe patients, but also because of a small number of these variants in patients with non-severe COVID-19. Since the burden of rare variants may be associated with chronic diseases (Ganna et al., 2018; Farhan et al., 2019; Xu et al., 2021; Zoghbi et al., 2021) and their severity and younger age of manifestation (Ganna et al., 2018), a genetic landscape with minimal numbers of rare variants may be associated with healthy aging and absence of comorbidities aggravating the course of COVID-19. Regarding comorbid conditions, overrepresentation of rare HI variants was found in all three subgroups of patients with severe COVID-19, with the lowest signal observed in the obese subgroup. The resulting differences may be largely random, but they may also reflect a greater contribution of a non-genetic component to the development of more severe disease in obese patients, as obesity reduces lung capacity and reserve, making ventilation difficult (Simonnet et al., 2020).

Intolerant genes are depleted by functional variation (HI and missense) in healthy populations and are subject to negative selection because they are associated with reduced fecundity (Gardner et al., 2022). In severe COVID-19 patients, an increased burden of QVs was observed in genes with specific cutoffs for intolerance to HI and missense variants. Selection of the most dangerous missense variants further strengthened the association signal. Because the results were obtained in independent strata of QVs, they can be considered confirming of each other in demonstrating the role of functional rare variants in severe COVID-19.

PID-related genes can be discussed as key genes for COVID-19 severity. In a recent systematic review involving data on 459 PID patients with COVID-19, the mortality rate was 9%, the hospitalization rate was 49%, and the oxygen use rate was 29% (Drzymalla et al., 2022), which is tens of times higher than in the general population (https://www.statista.com/statistics/1087466/covid19-cases-recoveries-deaths-worldwide/). Patients with cellular immunodeficiency and immune dysregulation may be more vulnerable to a severe disease course, but disease severity cannot be predicted for all people with PID, primarily because of the complex interplay between different immune branches (Delavari et al., 2021) and the incomplete penetrance and variable expressivity common in PID (Gruber and Bogunovic. 2020). Essential-for-life genes, which also showed an increased excess of rare HI variants in severe COVID-19, are enriched in genes intolerant to HI variants and disease genes (Dickinson et al., 2016), which are often overexpressed in disease-related tissues and exhibit high levels of network connectivity (Kolobkov et al., 2022). We can hypothesize that essential genes can be mutually regulated with more genes with fewer steps required to influence the core genes. Regarding the excess of rare HI variants in a number of disease genes, it should be noted that the first two most pronounced association signals were observed for genes of the immune and respiratory systems, i.e., systems predominantly involved in inflammation in SARS-CoV-2 infection (Pairo-Castineira, et al., 2021; Ramos-Casals et al., 2021).

The main limitation of the study is the rather small sample size, which may be subject to several types of bias. The test has insufficient power to identify candidate genes in gene-level burden tests to compare the results with those in the literature. We cannot rule out that association effects may be influenced by a limited number of high-penetrant variants in a subgroup of severe patients, but it is more likely that, in accordance with the ideas of the omnigenic model, the accumulation of weak effects of many rare functional variants throughout the exome contributes to the overall polygenic risk in patients with severe disease. This assumption is supported by biologically plausible data from additional analyses. The burden among rare and novel HI variants, missense, and synonymous variants exhibited a similar pattern, with the most significant signal found for novel HI variants. The greatest effect of damaging rare variants was observed for genes intolerant to these variants and potentially enriched for core COVID-19 genes (e.g., PID genes), and the smallest effect was found for genes that can be classified as peripheral (e.g., genes associated with neoplasia). However, a large number of genes cannot be strictly defined as core or peripheral genes, but rather can be characterized as some “intermediate” genes involved with greater or lesser efficiency in the regulatory modulation of disease-specific pathophysiological drivers.

In conclusion, our results are consistent with the hypothesis that small genetic effects of many rare variants contribute to the overall polygenic effect in severe COVID-19. Our study should be viewed as preliminary, suggesting a possible direction of research on COVID-19 phenotypes.

## Data Availability

All raw sequencing data from this study have been submitted to the NCBI BioProject database (https://www.ncbi.nlm.nih.gov/bioproject/) under accession number PRJNA947511.

## Supplementary material

SUPPLEMENTARY FIGURE S1 Characterization of variants from different AF bins. **(A)** Proportion of variants from different AF bins and different functional consequences in patients with severe and non-severe COVID-19. **(B)** Raincloud plots for HI, missense and synonymous variants exhibiting internal AF in different AF bins according to population databases. Each subplot for the AF bin includes cloud for core density data, umbrella for boxplot, rain for data points below the cloud, and flash for connecting lines between the mean values. The mean and SD of the internal AF for the AF bins are shown in the table. Raincloud plots were constructed using https://www.bioinformatics.com.cn/en, a free online platform for data analysis and visualization.

SUPPLEMENTARY FIGURE S2 Principal component analysis with 1,000 Genomes Project Phase 3 data. COVID-19 samples from our study (red) and 1,000 Genomes Phase 3 samples are plotted together based on principal components from overlapping SNP data. Five super populations: EUR, European; EAS, East Asian; AMR, Ad Mixed American; SAS, South Asian; AFR, African.

SUPPLEMENTARY FIGURE S3 Burden of rare HI, missense, and synonymous variants in 57 individuals with severe COVID-19 and 29 individuals with non-severe COVID-19. All singletons are excluded. Odds ratios and horizontal bars denoting 95% confidence intervals are shown. The number of variants/number of genes for each set of variants is shown in parentheses. The left subplot shows the distribution of variants per individual using a 100% stacked bar chart. The absolute numbers of variants per individual for each set of variants are indicated. Significant *p*-value threshold is 5.21 × 10^−4^.

SUPPLEMENTARY FIGURE S4 Burden of rare HI, missense, and synonymous variants in 52 individuals with severe COVID-19 and 27 individuals with non-severe COVID-19. Seven specimens identified as not belonging to the European superpopulation were excluded. Odds ratios and horizontal bars denoting 95% confidence intervals are shown. The number of variants/number of genes for each set of variants is shown in parentheses. The left subplot shows the distribution of variants per individual using a 100% stacked bar chart. The absolute numbers of variants per individual for each set of variants are indicated. Significant *p*-value threshold is 5.21 × 10^−4^.

## References

[B1] 1000 Genomes Project Consortium (2015). A global reference for human genetic variation. Nature 526, 68–74. 10.1038/nature15393 26432245 PMC4750478

[B2] Andrews S. (2010). FastQC: A quality control tool for high throughput sequence data. Available at: http://www.bioinformatics.babraham.ac.uk/projects/fastqc . [Accessed 01 September 2022]

[B3] Bailly L. Fabre R. Courjon J. Carles M. Dellamonica J. Pradier C. (2022). Obesity, diabetes, hypertension and severe outcomes among inpatients with coronavirus disease 2019: A nationwide study. Clin. Microbiol. Infect. 28, 114–123. 10.1016/j.cmi.2021.09.010 34537362 PMC8444420

[B4] Boyle E. A. Li Y. I. Pritchard J. K. (2017). An expanded view of complex traits: From polygenic to omnigenic. Cell 169, 1177–1186. 10.1016/j.cell.2017.05.038 28622505 PMC5536862

[B5] Brandi M. L. (2022). Are sex hormones promising candidates to explain sex disparities in the COVID-19 pandemic? Rev. Endocr. Metab. Disord. 23, 171–183. 10.1007/s11154-021-09692-8 34761329 PMC8580578

[B6] Brodin P. (2021). Immune determinants of COVID-19 disease presentation and severity. Nat. Med. 27, 28–33. 10.1038/s41591-020-01202-8 33442016

[B7] Butler-Laporte G. Povysil G. Kosmicki J. A. Cirulli E. T. Drivas T. Furini S. (2022). Exome-wide association study to identify rare variants influencing COVID-19 outcomes: Results from the Host Genetics Initiative. PLoS Genet. 18, e1010367. 10.1371/journal.pgen.1010367 36327219 PMC9632827

[B8] Casanova J. L. Su H. C. COVID Human Genetic Effort (2020). A global effort to define the human genetics of protective immunity to SARS-CoV-2 infection. Cell 181, 1194–1199. 10.1016/j.cell.2020.05.016 32405102 PMC7218368

[B9] Cingolani P. Patel V. M. Coon M. Nguyen T. Land S. J. Ruden D. M. (2012a). Using *Drosophila melanogaster* as a model for genotoxic chemical mutational studies with a new program. SnpSift. Front. Genet. 3, 35. 10.3389/fgene.2012.00035 22435069 PMC3304048

[B10] Cingolani P. Platts A. Wang le. L. Coon M. Nguyen T. Wang L. (2012b). A program for annotating and predicting the effects of single nucleotide polymorphisms, SnpEff: SNPs in the genome of *Drosophila melanogaster* strain w1118; iso-2; iso-3. Fly. (Austin) 6, 80–92. 10.4161/fly.19695 22728672 PMC3679285

[B11] Cirulli E. T. White S. Read R. W. Elhanan G. Metcalf W. J. Tanudjaja F. (2020). Genome-wide rare variant analysis for thousands of phenotypes in over 70,000 exomes from two cohorts. Nat. Commun. 11, 542. 10.1038/s41467-020-14288-y 31992710 PMC6987107

[B12] COVID-19 Host Genetics Initiative (2022). A first update on mapping the human genetic architecture of COVID-19. Nature 608, E1. 10.1038/s41586-022-04826-7 35922517 PMC9352569

[B13] COVID-19 Host Genetics Initiative (2021). Mapping the human genetic architecture of COVID-19. Nature 600, 472–477. 10.1038/s41586-021-03767-x 34237774 PMC8674144

[B14] D'Alterio G. Lasorsa V. A. Bonfiglio F. Cantalupo S. Rosato B. E. Andolfo I. (2022). Germline rare variants of lectin pathway genes predispose to asymptomatic SARS-CoV-2 infection in elderly individuals. Genet. Med. 24, 1653–1663. 10.1016/j.gim.2022.04.007 35511137 PMC9068606

[B15] Delavari S. Abolhassani H. Abolnezhadian F. Babaha F. Iranparast S. Ahanchian H. (2021). Impact of SARS-CoV-2 pandemic on patients with primary immunodeficiency. J. Clin. Immunol. 41, 345–355. 10.1007/s10875-020-00928-x 33263173 PMC7707812

[B16] Dickinson M. E. Flenniken A. M. Ji X. Teboul L. Wong M. D. White J. K. (2016). High-throughput discovery of novel developmental phenotypes. Nature 537, 508–514. 10.1038/nature19356 27626380 PMC5295821

[B17] Drzymalla E. Green R. F. Knuth M. Khoury M. J. Dotson W. D. Gundlapalli A. (2022). COVID-19-related health outcomes in people with primary immunodeficiency: A systematic review. Clin. Immunol. 243, 109097. 10.1016/j.clim.2022.109097 35973637 PMC9375253

[B18] Duman N. Tuncel G. Bisgin A. Bozdogan S. T. Sag S. O. Gul S. (2022). Analysis of ACE2 and TMPRSS2 coding variants as a risk factor for SARS-CoV-2 from 946 whole-exome sequencing data in the Turkish population. J. Med. Virol. 94, 5225–5243. 10.1002/jmv.27976 35811452 PMC9349697

[B19] Severe Covid-19 G WAS Group Ellinghaus D. Degenhardt F. Bujanda L. Buti M. Albillos A. (2020). Genomewide association study of severe covid-19 with respiratory failure. N. Engl. J. Med. 383, 1522–1534. 10.1056/NEJMoa2020283 32558485 PMC7315890

[B20] Evans L. Rhodes A. Alhazzani W. Antonelli M. Coopersmith C. M. French C. (2021). Executive summary: Surviving sepsis campaign: International guidelines for the management of sepsis and septic shock 2021. Crit. Care. Med. 49, 1974–1982. 10.1097/CCM.0000000000005357 34643578

[B21] Fallerini C. Picchiotti N. Baldassarri M. Zguro K. Daga S. Fava F. (2022). Common, low-frequency, rare, and ultra-rare coding variants contribute to COVID-19 severity. Hum. Genet. 141, 147–173. 10.1007/s00439-021-02397-7 34889978 PMC8661833

[B22] Farhan S. M. Howrigan D. P. Abbott L. E. Klim J. R. Topp S. D. Byrnes A. E. (2019). Exome sequencing in amyotrophic lateral sclerosis implicates a novel gene, DNAJC7, encoding a heat-shock protein. Nat. Neurosci. 22, 1966–1974. 10.1038/s41593-019-0530-0 31768050 PMC6919277

[B23] Feng Y. C. A. Howrigan D. P. Abbott L. E. Tashman K. Cerrato F. Singh T. (2019). Ultra-rare genetic variation in the epilepsies: A whole-exome sequencing study of 17,606 individuals. Am. J. Hum. Genet. 105, 267–282. 10.1016/j.ajhg.2019.05.020 31327507 PMC6698801

[B24] Force A. D. T. Ranieri V. M. Rubenfeld G. D. Thompson B. Ferguson N. Caldwell E. (2012). Acute respiratory distress syndrome. JAMA 307, 2526–2533. 10.1001/jama.2012.5669 22797452

[B25] Ganna A. Satterstrom F. K. Zekavat S. M. Das I. Kurki M. I. Churchhouse C. (2018). Quantifying the impact of rare and ultra-rare coding variation across the phenotypic spectrum. Am. J. Hum. Genet. 102, 1204–1211. 10.1016/j.ajhg.2018.05.002 29861106 PMC5992130

[B26] Gardner E. J. Neville M. D. Samocha K. E. Barclay K. Kolk M. Niemi M. E. (2022). Reduced reproductive success is associated with selective constraint on human genes. Nature 603, 858–863. 10.1038/s41586-022-04549-9 35322230

[B27] Giral H. Landmesser U. Kratzer A. (2018). Into the wild: GWAS exploration of non-coding RNAs. Front. Cardiovasc. Med. 5, 181. 10.3389/fcvm.2018.00181 30619888 PMC6304420

[B28] Gruber C. Bogunovic D. (2020). Incomplete penetrance in primary immunodeficiency: A skeleton in the closet. Hum. Genet. 139, 745–757. 10.1007/s00439-020-02131-9 32067110 PMC7275875

[B29] GTEx Consortium (2017). Genetic effects on gene expression across human tissues. Nature 550, 204–213. 10.1038/nature24277 29022597 PMC5776756

[B30] Iakoucheva L. M. Muotri A. R. Sebat J. (2019). Getting to the cores of autism. Cell 178, 1287–1298. 10.1016/j.cell.2019.07.037 31491383 PMC7039308

[B31] Ioannidis N. M. Rothstein J. H. Pejaver V. Middha S. McDonnell S. K. Baheti S. (2016). Revel: An ensemble method for predicting the pathogenicity of rare missense variants. Am. J. Hum. Genet. 99, 877–885. 10.1016/j.ajhg.2016.08.016 27666373 PMC5065685

[B32] Johnston J. J. Lewis K. L. Ng D. Singh L. N. Wynter J. Brewer C. (2015). Individualized iterative phenotyping for genome-wide analysis of loss-of-function mutations. Am. J. Hum. Genet. 96, 913–925. 10.1016/j.ajhg.2015.04.013 26046366 PMC4457956

[B33] Karczewski K. J. Francioli L. C. Tiao G. Cummings B. B. Alföldi J. Wang Q. (2020). The mutational constraint spectrum quantified from variation in 141,456 humans. Nature 581, 434–443. 10.1038/s41586-020-2308-7 32461654 PMC7334197

[B34] Kashatnikova D. A. Khadzhieva M. B. Kolobkov D. S. Belopolskaya O. B. Smelaya T. V. Gracheva A. S. (2022). Pneumonia and related conditions in critically ill patients-insights from basic and experimental studies. Int. J. Mol. Sci. 23, 9896. 10.3390/ijms23179896 36077293 PMC9456259

[B35] Kolobkov D. S. Sviridova D. A. Abilev S. K. Kuzovlev A. N. Salnikova L. E. (2022). Genes and diseases: Insights from transcriptomics studies. Genes (Basel) 13, 1168. 10.3390/genes13071168 35885950 PMC9317567

[B36] Kosmicki J. A. Horowitz J. E. Banerjee N. Lanche R. Marcketta A. Maxwell E. (2021b). Catalog of associations between rare coding variants and COVID-19 outcomes. medRxiv [Preprint]. 10.1101/2020.10.28.20221804

[B37] Kosmicki J. A. Horowitz J. E. Banerjee N. Lanche R. Marcketta A. Maxwell E. (2021a). Pan-ancestry exome-wide association analyses of COVID-19 outcomes in 586,157 individuals. Am. J. Hum. Genet. 108, 1350–1355. 10.1016/j.ajhg.2021.05.017 34115965 PMC8173480

[B38] Kwok A. J. Mentzer A. Knight J. C. (2021). Host genetics and infectious disease: New tools, insights and translational opportunities. Nat. Rev. Genet. 22, 137–153. 10.1038/s41576-020-00297-6 33277640 PMC7716795

[B39] Lek M. Karczewski K. J. Minikel E. V. Samocha K. E. Banks E. Fennell T. (2016). Analysis of protein-coding genetic variation in 60,706 humans. Nature 536, 285–291. 10.1038/nature19057 27535533 PMC5018207

[B40] Li H. (2013). Aligning sequence reads, clone sequences and assembly contigs with BWA-MEM. arXiv preprint arXiv:1303.3997.

[B41] Lombardo M. V. Pramparo T. Gazestani V. Warrier V. Bethlehem R. A. Carter Barnes C. (2018). Large-scale associations between the leukocyte transcriptome and BOLD responses to speech differ in autism early language outcome subtypes. Nat. Neurosci. 21, 1680–1688. 10.1038/s41593-018-0281-3 30482947 PMC6445349

[B42] López-Rodríguez R. Del Pozo-Valero M. Corton M. Minguez P. Ruiz-Hornillos J. Pérez-Tomás M. E. (2022). Presence of rare potential pathogenic variants in subjects under 65 years old with very severe or fatal COVID-19. Sci. Rep. 12, 10369. 10.1038/s41598-022-14035-x 35725860 PMC9208539

[B43] MacArthur D. G. Balasubramanian S. Frankish A. Huang N. Morris J. Walter K. (2012). A systematic survey of loss-of-function variants in human protein-coding genes. Science 335, 823–828. 10.1126/science.1215040 22344438 PMC3299548

[B44] Marouli E. Graff M. Medina-Gomez C. Lo K. S. Wood A. R. Kjaer T. R. (2017). Rare and low-frequency coding variants alter human adult height. Nature 542, 186–190. 10.1038/nature21039 28146470 PMC5302847

[B45] Mathieson I. (2021). The omnigenic model and polygenic prediction of complex traits. Am. J. Hum. .Genet. 108, 1558–1563. 10.1016/j.ajhg.2021.07.003 34331855 PMC8456163

[B46] McDonald J. H. (2009). Handbook of biological statistics. Baltimore: Sparky House Publishing.

[B47] Nhung V. P. Ton N. D. Ngoc T. T. B. Thuong M. T. H. Hai N. T. T. Oanh K. T. P. (2022). Host genetic risk factors associated with COVID-19 susceptibility and severity in Vietnamese. Genes (Basel) 13, 1884. 10.3390/genes13101884 36292769 PMC9601961

[B48] O’Driscoll M. Ribeiro Dos Santos G. Wang L. Cummings D. A. Azman A. S. Paireau J. (2021). Age-specific mortality and immunity patterns of SARS-CoV-2. Nature 590, 140–145. 10.1038/s41586-020-2918-0 33137809

[B49] Pairo-Castineira E. Clohisey S. Klaric L. Bretherick A. D. Rawlik K. Pasko D. (2021). Genetic mechanisms of critical illness in COVID-19. Nature 591, 92–98. 10.1038/s41586-020-03065-y 33307546

[B80] Peckham H. de Gruijter N. M. Raine C. Radziszewska A. Ciurtin C. Wedderburn L. R. (2020). Male sex identified by global COVID-19 meta-analysis as a risk factor for death and ITU admission. Nat. Commun. 11, 6317. 10.1038/s41467-020-19741-6 33298944 PMC7726563

[B50] Petersen D. C. Steyl C. Scholtz D. Baker B. Abdullah I. Uren C. (2022). African genetic representation in the context of SARS-CoV-2 infection and COVID-19 severity. Front. Genet. 13, 909117. 10.3389/fgene.2022.909117 35620464 PMC9127354

[B51] Petrovski S. Todd J. L. Durheim M. T. Wang Q. Chien J. W. Kelly F. L. (2017). An exome sequencing study to assess the role of rare genetic variation in pulmonary fibrosis. Am. J. Respir. Crit. Care Med. 196, 82–93. 10.1164/rccm.201610-2088OC 28099038 PMC5519963

[B52] Pullabhatla V. Roberts A. L. Lewis M. J. Mauro D. Morris D. L. Odhams C. A. (2018). De novo mutations implicate novel genes in systemic lupus erythematosus. Hum. Mol. Genet. 27, 421–429. 10.1093/hmg/ddx407 29177435 PMC5886157

[B53] Rahardja D. Yang Y. Zhang Z. (2016). A comprehensive review of the two-sample independent or paired binary data, with or without stratum effects. J. Mod. Appl. Stat. Methods 15, 16. 10.22237/jmasm/1478002440

[B54] Ramos-Casals M. Brito-Zerón P. Mariette X. (2021). Systemic and organ-specific immune-related manifestations of COVID-19. Nat. Rev. Rheumatol. 17, 315–332. 10.1038/s41584-021-00608-z 33903743 PMC8072739

[B55] Salnikova L. E. Khadzhieva M. B. Kolobkov D. S. Gracheva A. S. Kuzovlev A. N. Abilev S. K. (2020). Cytokines mapping for tissue-specific expression, eQTLs and GWAS traits. Sci. Rep. 10, 14740. 10.1038/s41598-020-71018-6 32895400 PMC7477549

[B56] Salzberger B. Buder F. Lampl B. Ehrenstein B. Hitzenbichler F. Holzmann T. (2021). Epidemiology of SARS-CoV-2. Infection 49, 233–239. 10.1007/s15010-020-01531-3 33034020 PMC7543961

[B57] Scelsi M. A. Napolioni V. Greicius M. D. Altmann A. Alzheimer’s Disease Neuroimaging Initiative (Adni) and the Alzheimer’s Disease Sequencing Project (Adsp) (2021). Network propagation of rare variants in Alzheimer's disease reveals tissue-specific hub genes and communities. PLoS Comput. Biol. 7, e1008517. 10.1371/journal.pcbi.1008517 PMC7817020 33411734

[B58] Shcherbak S. G. Changalidi A. I. Barbitoff Y. A. Anisenkova A. Y. Mosenko S. V. Asaulenko Z. P. (2022). Identification of genetic risk factors of severe COVID-19 using extensive phenotypic data: A proof-of-concept study in a cohort of Russian patients. Genes (Basel) 13, 534. 10.3390/genes13030534 35328087 PMC8949130

[B59] Silk M. Petrovski S. Ascher D. B. (2019). MTR-viewer: Identifying regions within genes under purifying selection. Nucleic Acids Res. 47, W121–W126. 10.1093/nar/gkz457 31170280 PMC6602522

[B60] Simonnet A. Chetboun M. Poissy J. Raverdy V. Noulette J. Duhamel A. (2020). High prevalence of obesity in severe acute respiratory syndrome coronavirus-2 (SARS-CoV-2) requiring invasive mechanical ventilation. Obes. (Silver Spring) 28, 1195–1199. 10.1002/oby.22831 PMC7262326 32271993

[B61] Sokologorskiy S. V. Ovechkin A. M. Khapov I. V. Politov M. E. Bulanova E. L. (2022). Risk factors of severe disease and methods for clinical outcome prediction in patients with COVID-19 (Review). Obshchaya Reanimatol. = General Reanimatol. 18, 31–38. 10.15360/1813-9779-2022-1-31-38

[B62] Takahashi T. Ellingson M. K. Wong P. Israelow B. Lucas C. Klein J. (2020). Sex differences in immune responses that underlie COVID-19 disease outcomes. Nature 588, 315–320. 10.1038/s41586-020-2700-3 32846427 PMC7725931

[B63] Taliun D. Harris D. N. Kessler M. D. Carlson J. Szpiech Z. A. Torres R. (2021). Sequencing of 53,831 diverse genomes from the NHLBI TOPMed program. Nature 590, 290–299. 10.1038/s41586-021-03205-y 33568819 PMC7875770

[B64] Talla A. Vasaikar S. V. Lemos M. P. Moodie Z. Pebworth M. P. L. Henderson K. E. (2021). Longitudinal immune dynamics of mild COVID-19 define signatures of recovery and persistence. bioRxiv [Preprint]. 10.1101/2021.05.26.442666

[B65] Tangye S. G. Al-Herz W. Bousfiha A. Cunningham-Rundles C. Franco J. L. Holland S. M. (2022). Human inborn errors of immunity: 2022 update on the classification from the international union of immunological societies expert committee. J. Clin. Immunol. 42, 1473–1507. 10.1007/s10875-022-01289-3 35748970 PMC9244088

[B66] Van der Auwera G. A. O'Connor B. D. (2020). Genomics in the cloud: Using docker, GATK, and WDL in terra. 1st Edition. Sebastopol, CA: O'Reilly Media.

[B67] Vuckovic D. Bao E. L. Akbari P. Lareau C. A. Mousas A. Jiang T. (2020). The polygenic and monogenic basis of blood traits and diseases. Cell 182, 1214–1231. 10.1016/j.cell.2020.08.008 32888494 PMC7482360

[B68] Webb Hooper M. Nápoles A. M. Pérez-Stable E. J. (2020). COVID-19 and racial/ethnic disparities. JAMA 323, 2466–2467. 10.1001/jama.2020.8598 32391864 PMC9310097

[B69] Webb K. Peckham H. Radziszewska A. Menon M. Oliveri P. Simpson F. (2019). Sex and pubertal differences in the type 1 interferon pathway associate with both X chromosome number and serum sex hormone concentration. Front. Immunol. 9, 3167. 10.3389/fimmu.2018.03167 30705679 PMC6345344

[B70] World Health Organization (2020). Clinical management of COVID-19: Interim guidance. Geneva, Switzerland: World Health Organization.

[B71] Wray N. R. Wijmenga C. Sullivan P. F. Yang J. Visscher P. M. (2018). Common disease is more complex than implied by the core gene omnigenic model. Cell 173, 1573–1580. 10.1016/j.cell.2018.05.051 29906445

[B72] Xu H. Zhen Q. Bai M. Fang L. Zhang Y. Li B. (2021). Deep sequencing of 1320 genes reveals the landscape of protein-truncating variants and their contribution to psoriasis in 19,973 Chinese individuals. Genome Res. 31, 1150–1158. 10.1101/gr.267963.120 34155038 PMC8256863

[B73] Yang J. Zheng Y. A. Gou X. Pu K. Chen Z. Guo Q. (2020). Prevalence of comorbidities and its effects in patients infected with SARS-CoV-2: A systematic review and meta-analysis. Int. J. Infect. Dis. 94, 91–95. 10.1016/j.ijid.2020.03.017 32173574 PMC7194638

[B74] Zeng Z. Bromberg Y. (2019). Predicting functional effects of synonymous variants: A systematic review and perspectives. Front. Genet. 10, 914. 10.3389/fgene.2019.00914 31649718 PMC6791167

[B75] Zguro K. Fallerini C. Fava F. Furini S. Renieri A. (2022). Host genetic basis of COVID-19: From methodologies to genes. Eur. J. Hum. Genet. 30, 899–907. 10.1038/s41431-022-01121-x 35618891 PMC9135575

[B76] Zhang Q. Bastard P. Liu Z. Le Pen J. Moncada-Velez M. Chen J. (2020). Inborn errors of type I IFN immunity in patients with life-threatening COVID-19. Science 370, eabd4570. 10.1126/science.abd4570 32972995 PMC7857407

[B77] Zhou H. Arapoglou T. Li X. Li Z. Zheng X. Moore J. (2023). Favor: Functional annotation of variants online resource and annotator for variation across the human genome. Nucleic Acids Res. 51, D1300. 10.1093/nar/gkac966 36350676 PMC9825437

[B78] Zhu X. Bai W. Zheng H. (2021). Twelve years of GWAS discoveries for osteoporosis and related traits: Advances, challenges and applications. Bone Res. 9, 23. 10.1038/s41413-021-00143-3 33927194 PMC8085014

[B79] Zoghbi A. W. Dhindsa R. S. Goldberg T. E. Mehralizade A. Motelow J. E. Wang X. (2021). High-impact rare genetic variants in severe schizophrenia. Proc. Natl. Acad. Sci. U.S.A. 118, e2112560118. 10.1073/pnas.2112560118 34903660 PMC8713775

