# Supplementary figures and images for: COVID-19 severity: does the genetic landscape of rare variants matter?
Source: Front Genet. 2023 Jun 29;14:1152768. doi: 10.3389/fgene.2023.1152768 (PMC10339319; doi:10.3389/fgene.2023.1152768)

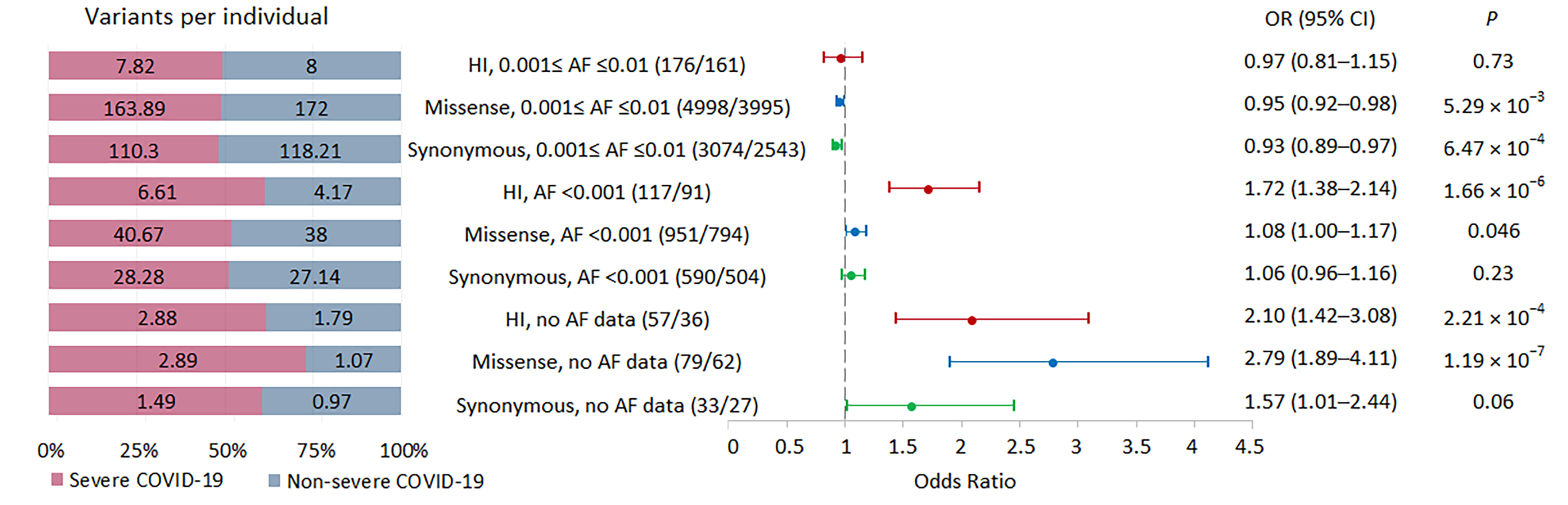

Supplement: Supplementary file 1 [file Image3.TIF]

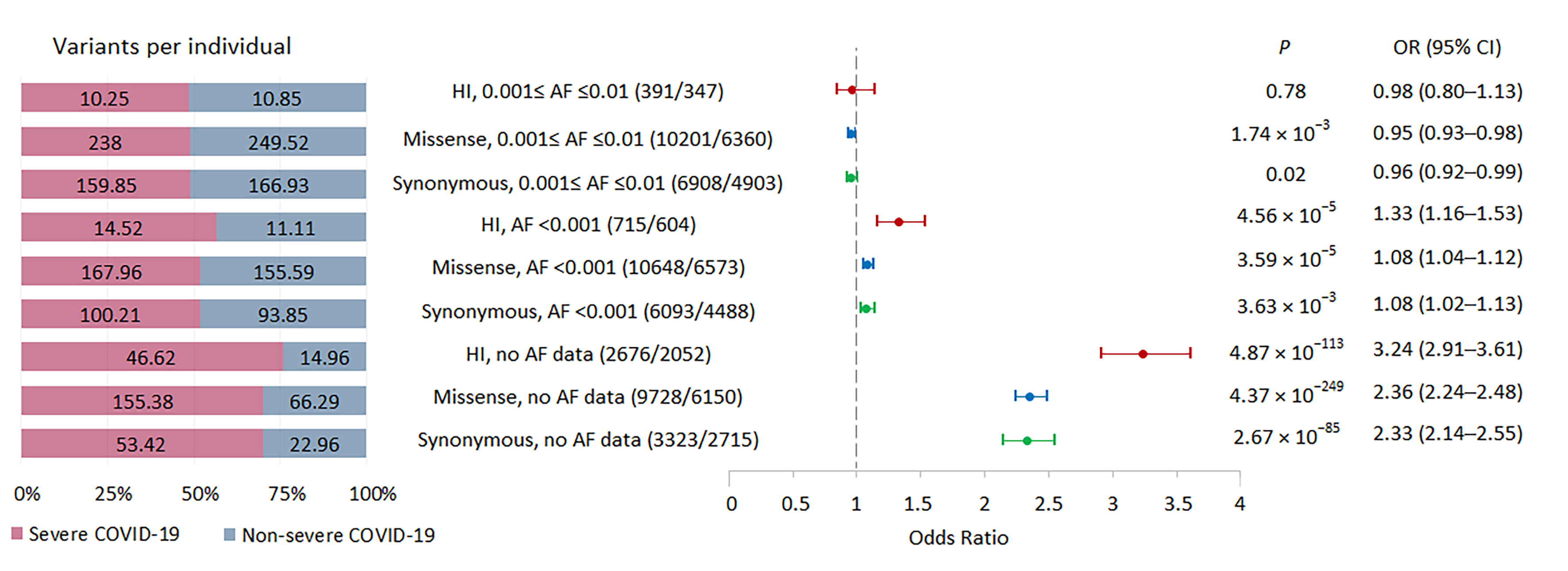

Supplement: Supplementary file 2 [file Image4.TIF]

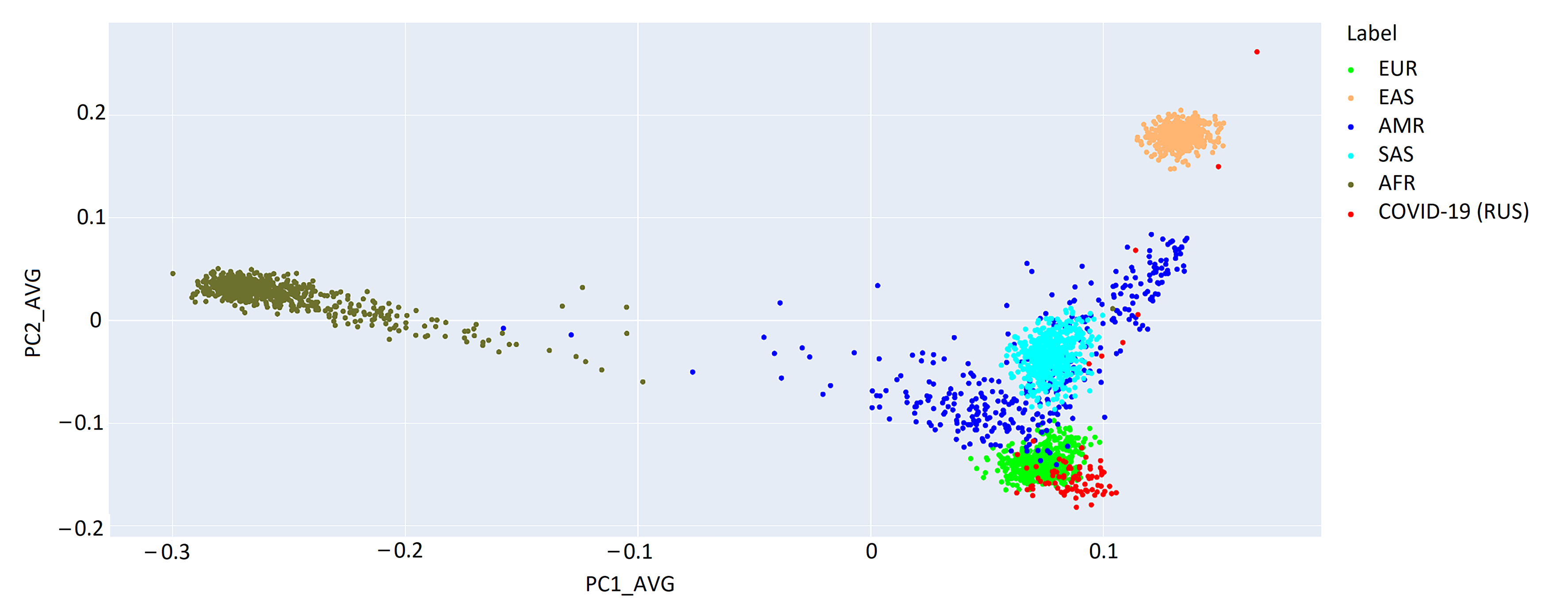

Supplement: Supplementary file 3 [file Image2.TIF]

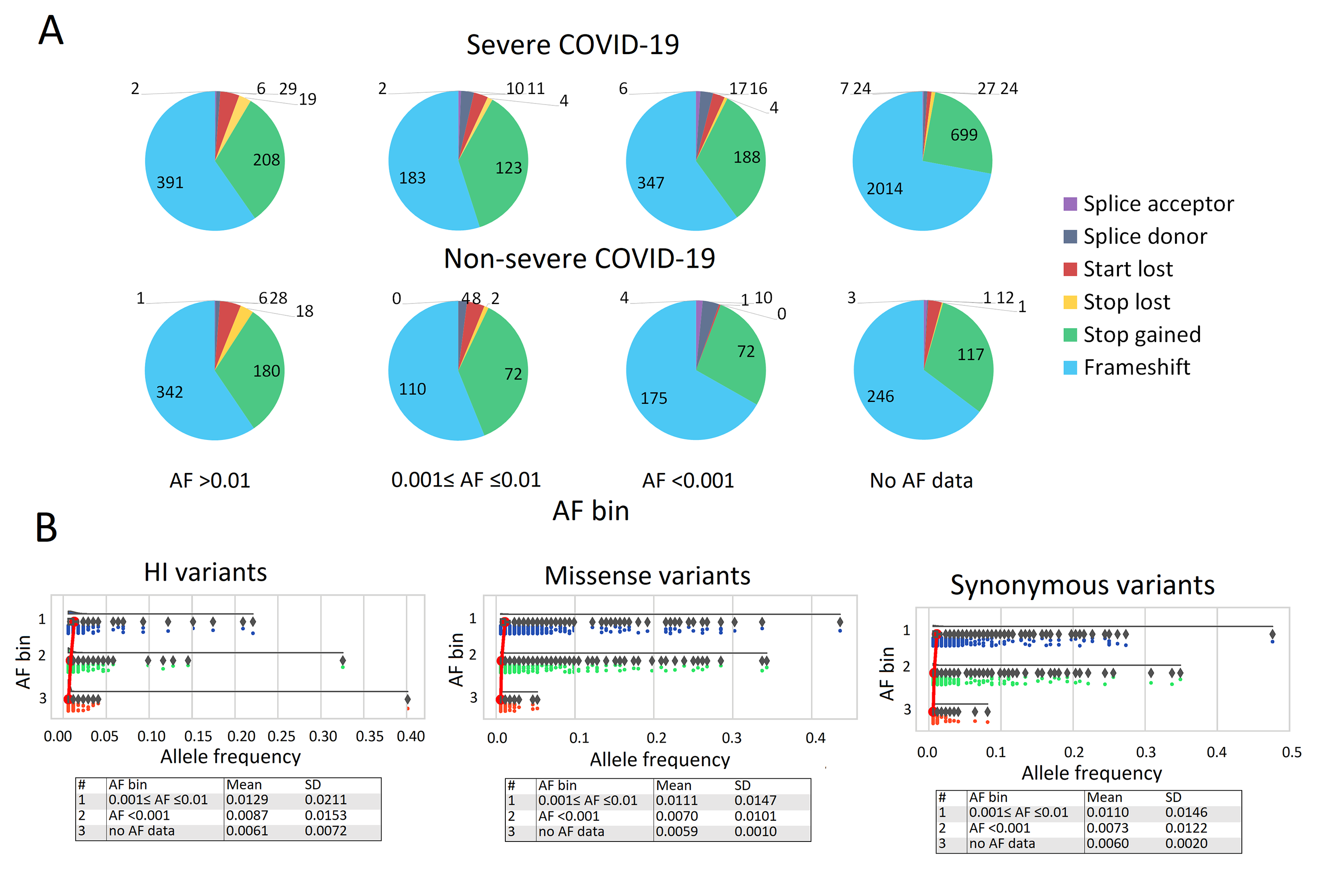

Supplement: Supplementary file 4 [file Image1.TIF]
